# Draft Genome Sequences of Three Bacterial Isolates from Cultures of the Marine Diatom *Thalassiosira rotula*

**DOI:** 10.1128/genomeA.00316-17

**Published:** 2017-05-04

**Authors:** Nathan S. Garcia, Cheuk-Man Yung, Katherine M. Davis, Tatiana Rynearson, Dana E. Hunt

**Affiliations:** a Division of Marine Sciences and Conservation, Duke University, Beaufort, North Carolina, USA; b Graduate School of Oceanography, University of Rhode Island, Narragansett, Rhode Island, USA

## Abstract

Phytoplankton often both provision and depend on heterotrophic bacteria. In order to investigate these relationships further, we sequenced draft genomes of three bacterial isolates from cultures of the marine diatom *Thalassiosira rotula* to identify metabolic functions that may support interactions with *T. rotula*.

## GENOME ANNOUNCEMENT

The natural habitat of some marine bacteria may not be oligotrophic ocean waters but, rather, chemically distinct microenvironments associated with algal cells or other particle types (1–3). In this study, we specifically focus on interactions between phytoplankton and heterotrophic bacteria, as heterotrophic bacteria influence the productivity and transcription of their algal partners through nutrient remineralization, vitamin production, hydrogen peroxide scavenging, and small molecule signaling (4, 5). In return, the bacteria benefit from oxygen and organic material produced by photoautotrophs and potentially preferentially feed on specific phytoplankton-derived compounds (6). Here, we investigate bacteria isolated from cultures of the cosmopolitan marine diatom *Thalassiosira rotula* (7).

Heterotrophs were isolated from two strains of *T. rotula* (CCMP3264, CCMP3362) on F/2 (8) supplemented with 0.1 g L^−1^ yeast extract; cultures were then frozen (−80°C) until genomic DNA extraction using the Gentra Puregene yeast/bacteria kit (QIAGEN). We sequenced genomes from three isolates chosen based on the prevalence of their 16S rRNA gene sequences in xenic cultures of *T. rotula*: the gammaproteobacterial strains *Alteromonas* sp. W12 (from *T. rotula* strain CCMP3362) and *Marinobacter* sp. C18 (from *T. rotula* strain CCM3264) and the alphaproteobacterium *Maricaulis* sp. W15 (from *T. rotula* strain CCMP3362).

For each of the strains, 500-bp insert TruSeq libraries were sequenced with the HiSeq2000 platform (Illumina) using 125-bp paired-end reads at the Duke Center for Genomic and Computational Biology, Durham, NC, USA; 43, 48, and 41 million paired-end reads with an average length of 126 bp were generated for *Alteromonas* sp. W12, *Marinobacter* sp. C18, and *Maricaulis* sp. W15, respectively. The FASTX trimmer was used to remove low-quality reads by trimming 5 bases off the 5′ end and 10 bases off the 3′ end. Reads were assembled using SPAdes version 3.6.1 with read error correction (9) and assessed with QUAST version 3.0 (10). We obtained 14, 42, and 22 contigs (>2 kb) and total draft genomes of lengths 4.6, 4.9, and 3.5 Mb for *Alteromonas* sp. W12, *Marinobacter* sp. C18, and *Maricaulis* sp. W15, respectively, which were then annotated with the NCBI Prokaryotic Genome Annotation Pipeline (11). This process identified 3,958, 4,566, and 3,297 genes for *Alteromonas* sp. W12, *Marinobacter* sp. C18, and *Maricaulis* sp. W15, respectively.

To identify potential cross-domain interactions between these strains and *T. rotula*, we used BLASTp to search the bacterial genomes (E value <10^−20^) for genes that may mediate these processes. Many phytoplankton benefit from vitamins produced by bacteria, and each of these isolates contained genes involved in vitamin B_1_, B_7_, or B_12_ synthesis (12). All heterotrophic isolates also contain catalase genes, which can reduce phytoplankton oxidative stress via hydrogen peroxide scavenging (4). Additionally, organic sulfur-containing compounds produced by phytoplankton may indicate a specific phytoplankton-associated habitat: while the first gene in the pathway for dihydroxypropane-1-sulfonate degradation (6) is present in all isolates, dimethylsulfoniopropionate demethylase (13) was found only in the *Marinobacter* sp. C18 strain. Understanding the distribution of genes potentially mediating phytoplankton–heterotroph interactions is important in determining how these processes may affect biogeochemical cycling.

### Accession number(s).

The draft genome sequences reported here were deposited in NCBI GenBank under the accession numbers LQXH01000012 (*Alteromonas* sp. W12), LQXJ00000000 (*Marinobacter* sp. C18), and LQXG00000000 (*Maricaulis* sp. W15).
